# Single dose targeted intraoperative radiotherapy (TARGIT) for breast  cancer  can be delivered as a second procedure under local anaesthetic

**DOI:** 10.1186/1477-7819-4-2

**Published:** 2006-01-17

**Authors:** Jayant S Vaidya, Lynn Walton, John Dewar

**Affiliations:** 1 Departments of Surgery & Molecular Oncology, Level 6, Ninewells Hospital and Medical School, University of Dundee, Dundee, DD1 9SY, UK; 2 Department of Anaesthesia, Level 6, Ninewells Hospital and Medical School, University of Dundee, Dundee, DD1 9SY, UK; 3 Department of Clinical Oncology, Level 6, Ninewells Hospital and Medical School, University of Dundee, Dundee, DD1 9SY

## Abstract

**Background:**

Intraoperative radiotherapy (IORT) is promising approach that is being tested in randomised clinical trials. In the Targit (TARGeted Intraopeartive radioTherapy) trial IORT can be delivered at the time of primary surgery or as a second procedure. Patients prefer the single procedure of intraoperative radiotherapy even if it is under general anaesthetic to 6-weeks of daily visits for conventional external beam radiotherapy.

**Case presentation:**

We report a case of a 70 year lady who underwent lumpectomy and axillary sampling and in whom we successfully administered IORT under local anaesthetic.

**Conclusion:**

In selected patients, this attractive option may make the procedure even more widely applicable.

## Background

Partial breast irradiation for breast cancer is being tested in randomised trials[1]. In the TARGIT trial [2] patients can receive the TARGeted Intraoperative radioTherapy either during the primary operation or as a second procedure. Over 300 patients have been randomised in this multi-centre trial to date.

## Case presentation

A 70 year old lady who had a lumpectomy and axillary sample for a 2.8 cm, grade II, node-negative breast cancer was with her consent, entered into the trial. She was randomised to receive intraoperative radiotherapy as a second procedure. However, the attending anaesthetist felt that because of the history of severe ischaemic heart disease a second general anaesthetic should be avoided. The patient was keen to take the intraoperative radiotherapy to which she was randomised, rather than come every day for radiotherapy for 6 weeks; she was willing to undergo the whole procedure under local anaesthetic.

She received temazepam (10 mg), preoperatively. She was given midazolam 1 mg i.v. just prior to infiltration of the scar (just the skin) with 1% lignocaine with 1:200,000 adrenaline and 0.5% marcaine in a ration of 1:1. During the infiltration she breathed a 50:50 mixture of oxygen and nitrous oxide, followed by oxygen @ 6 L/min via Hudson mask.

**Figure 1:**
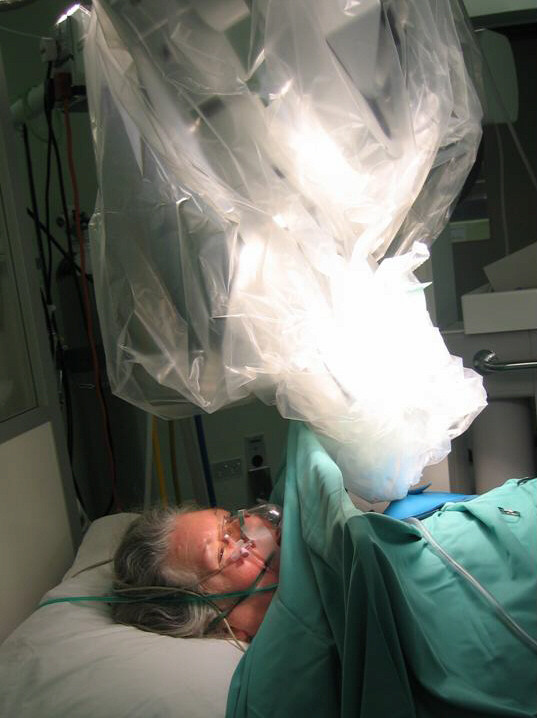
The patient (eyes open) and the Intrabeam device. The breast is covered with lead shield (blue). [the patient has given explicit permission for this photograph to be published]

**Figure 2:**
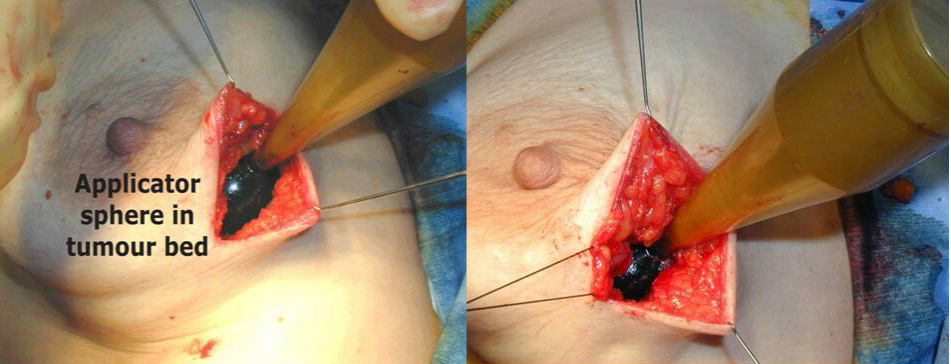
The spherical applicator is inserted in the tumour bed and the target breast tissue is wrapped around with a purse string.

The cavity was opened and radiotherapy applicator positioned with a purse string suture as usual. The patient did not feel any pain and tolerated the procedure well (figure 1). She remained still during the 22 minutes while radiotherapy (20 Gy at surface) was delivered to the tumour bed (figure 2). Postoperative period was uneventful and we discharged home a happy patient.

## Discussion

Delivering intraoperative radiotherapy ("targiting") as a second procedure allows proper selection of patients because detailed histopathology is available. The approach can also extend the applicability of intraoperative radiotherapy to patients who have already had operation in other centres.  The Targit technique with Intrabeam^(TM)^ delivers radiotherapy  from within the tumour bed and does not require extensive dissection of  breast tissue; it is therefore feasible under a local anaesthetic. If the Targit trial is successful, it is likely that many suitable patients will have significant co-morbidities and a second general anaesthetic would be ideally avoided. It appears that this is possible in selected patients.

## Competing interests

The author(s) declare that they have no competing interests.
